# Expanding Occupational Therapy Perspectives with Family Caregivers

**DOI:** 10.1177/00084174221103952

**Published:** 2022-09-22

**Authors:** Louise Demers

**Keywords:** Client-professional relationship, Family caregivers, Frames of reference, Occupational therapy, Scope of practice, Strength-based practice

## Abstract

**Background**. Family caregivers are ever-present and crucial collaborators in the work of occupational therapists but are rarely the focus of their efforts. **Purpose**. This lecture will discuss the greater inclusion of family caregivers in occupational therapy and the exciting possibilities that emerge from this change. **Key issues**. Family caregivers are a unique client population. This position statement is supported by recent research on occupational therapists’ values and shifts towards an occupational participation approach in the profession. Working with this client population requires a nuanced understanding of their experience. Caregiving can be burdensome, but it can also create positive effects many of which can be identified and understood through a relational lens. **Implications**. Through a three-fold approach, occupational therapists can work with caregivers to locate and mitigate negative caregiving effects, discover, and build on positive effects, and further develop positive outcomes by encouraging and balancing caregiving and non-caregiving occupations.

## Introduction

Throughout most of my research career, my passion has been two-fold: the first line of inquiry has been to analyze and measure different occupational phenomena to monitor and assess change over time. Working alongside many talented people I have tried to understand and create tools to measure different types of concepts important in occupational therapy (OT) and in rehabilitation. The second line of research that has dominated my career is gerontechnology, focusing on measuring the needs and impact of different assistive technologies on users and caregivers. In recent years, I have been honored to co-lead a work package with AGEWELL, which is Canada’s technology and aging network (https://agewell-nce.ca/). During my time in this role, ever-present and crucial collaborators in the work of occupational therapists (OTs) captured my imagination. Oftentimes, these collaborators are indispensable to our work but are rarely the focus of our efforts. These collaborators are family caregivers.

A family caregiver is defined as a family member, friend, or another person who provides voluntarily or involuntarily physical or emotional care to a person experiencing mental or physical health issues or a person with disabilities (Keating et al., 2019). These caregivers often provide unpaid labor to people with whom they have some sort of relationship prior to the care episode. This lecture will focus on, what I see as, the changing role of family caregivers in OT and the exciting possibilities that emerge from this change.

I will begin my lecture with a discussion of the central role that family caregivers play in our work, our society, and, likely, in our personal lives. Next, I propose considering caregivers as a unique client population and show how approaching them as clients fits with OT values and theory. I will then discuss how conceptualizing an approach to caregivers-as-clients can move beyond a caregiver burden perspective by considering an aspect of their experience that I believe deserves greater attention, that is the positive effects of caregiving. Finally, I conclude the lecture by introducing a caregiver-specific approach to working with them, both as care team partners and as clients.

At its core, my lecture is a call to clinicians and researchers alike to deepen our understanding of, and interaction with, caregivers in OT practice, research, and theory. I invite all to see the caregiver not only as a key member of a care team who is instrumental in intervention efficacy but also as a client in their own rights who survives and thrives in all their occupations.

## Why Caregivers Matter

### An Illustrative Story

I would like to begin by sharing an illustrative story from my personal life. It was one of many moments in recent years that highlighted for me the need for a new approach to caregivers-as-clients.

A few years ago, my 88-year-old father-in-law, Hubert, started to deteriorate in terms of his health and participation. He had survived multiple coronary bypasses and was adapting to living with sarcopenia, memory loss, hearing loss, and musculoskeletal pain. Over time, he was less able to prepare meals, he started sleeping excessively, and he was losing interest in social activities. It was becoming clear that Hubert needed and wanted extra help with accomplishing some everyday tasks. My partner and I became family caregivers.

For me, this was the first time that I had taken on this role for a person other than my own children. After several months, it became increasingly difficult to juggle providing care to Hubert and meeting the demands of our jobs. Thankfully, we were able to set up home care services for assistance during the week with tasks such as housekeeping, morning care, medication, and two meals a day. On the weekends, my partner and I would take over performing some of these tasks. Together, Hubert, my partner, the family, the care support, and myself, worked to achieve relative stability in our new routines.

Throughout this caregiving episode, I felt a wide range of emotions. During the week, my partner and I found ourselves worrying about Hubert. Every unexpected phone call made us jump: was it bad news? If we called and he didn’t answer, we worried about his welfare: did something happen? Did he fall? Was he okay? We felt frustration and sometimes a sense of guilt for not being able to be more present. On weekends, I sometimes found myself emotionally and physically exhausted by some caregiving tasks and distressed by Hubert’s continued decline. Yet, I felt grateful and rewarded by a deepening relationship with Hubert and with my partner too. At the end of his life, Hubert was still able to say how important we were to him. I was deeply saddened, and somewhat relieved, when he eventually passed away last summer at the beginning of the COVID pandemic. Having had the chance to care for him and hear him express how important our years of caregiving were to him, still means so much to me.

As I share this personal story, I am very aware of its apparent ordinariness. My personal experience of family caregiving was not necessarily unique, nor was it long or particularly challenging, but that is the point. I share my experience of family caregiving to highlight the idea that caring for a family member or friend is almost a universal role that most of us, if we have not already, will take on in our lives, and quite possibly multiple times. The ubiquitous nature of family caregiving is the fundamental reason why OT should continue to deepen its understanding of and interactions with caregivers.

### We Are All Caregivers (or Will Be)

The profile and contribution of family caregivers to Canadian society are dynamic and profound. In the 2018 census, one in four Canadians aged 15 or older reported providing care to a family member or friend with a long-term health condition, disability, or age-related need (Harding & Omercic, 2020; Statistics Canada, 2020). Of the roughly 7.8 million family caregivers in Canada, about 54% identified as women, 47%, like myself, cared for a parent or parent-in-law, 13% cared for a spouse or partner, and 13% indicated that they cared for a friend, neighbor, or an acquaintance (Harding & Omercic, 2020; Statistics Canada, 2020).

As you might expect, the economic and social contribution of family caregivers’ labor is significant in Canadian society, especially in our healthcare systems. In fact, our healthcare systems across the country have historically relied heavily on family caregivers (Reinhard et al., 2019) and this reliance is only escalating over time as patients are released from the hospital earlier and caregivers are increasingly asked to perform complex caring tasks at home (Johnson & Bacsu, 2018).

The economic contribution and savings to healthcare systems that caregivers provide are truly invaluable. Some, however, have tried to quantify this. For example, in Quebec, it is estimated that 1.9 million family caregivers contribute 743 million hours of unpaid labor per year. This, in turn, saves Santé Québec approximately 9.3 billion dollars per year (Eales, Kim, Riopel, et al., 2020). And this is only Quebec. In Alberta, it is estimated that 388 million hours of unpaid caregiving labor performed per year by nearly 1 million caregivers save Alberta Health Services 5.8 billion dollars per year (Eales, Kim, Sereda, and Fast, 2019). A similar story can be told in every province and territory across Canada (see Eales, Kim, Campbell, et al., 2020 and Eales, Kim, Riopel, et al., 2020 for further examples). Yet, despite the sheer number of caregivers and the massive contributions they make to our society, very few caregivers are trained for this role.

Caregivers can struggle to find a balance between the different occupations in their lives—such as paid employment, other commitments to family, friends, or community, or finding time for leisure activities and relaxation. Without them, however, many of the positive outcomes that the care recipient achieves could be compromised, and in some cases, not possible at all.

### OTs Need Caregivers

As OTs, we are acutely aware of the integral and challenging role that family caregivers play in the lives of people with long-term health conditions, disabilities, or age-related needs. From the early days of our training, we are taught to understand the complexities of family dynamics that surround the patient and their care (Moghimi, 2007). The caregiver is thus included in the models that shape OT practice and research. For example, the Canadian Model of Occupational Performance and Engagement conceptualizes the caregiver as one of the four environmental factors that influence a person and their occupation (Polatajko et al., 2007). The Human Occupational Model situates “people” (including family and friend caregivers) as input and feedback from the care recipient’s environment (Kielhofner & Posatery Burke, 1980). And finally, the Person-Environment-Occupation-Performance model also positions family caregivers in the realm of the care recipient’s “environment” as social support, alongside other factors such as social and economic systems, culture and values, technologies, and the built and natural environment (Baum et al., 2015).

I am certain that these models resonate with the experiences of many OT clinicians and researchers. We can likely all attest that caregivers are an important environmental factor or influence in our work. Caregivers play a vital role in implementing recommendations that improve the lives of care recipients. Indeed, researchers have noted that gains made during rehabilitation can be more effectively maintained when the family members are supportive and involved (Evans et al., 1994).

Thus, it could be said that OTs need caregivers to assist with a wide variety of tasks that are central to our action plan with the care recipient. These tasks can include managing medications, helping with mobility and transfers, advocating for the healthcare needs of the care recipient, and the list goes on and on. Without caregivers, the work of OTs would likely be less impactful.

### Caregivers Need OTs

Likewise, as OTs need caregivers, many caregivers would likely agree that there is a need for OTs in their lives as well. While caregivers help accomplish the aims of our care recipient-centered interventions, many of our interventions improve and facilitate the care they provide. For example, we educate and train caregivers regarding how to perform caregiving tasks, provide opportunities to practice new care-related skills, and give feedback and coaching (Moghimi, 2007). We can also improve the health literacy and the skills of caregivers by showing them how to use assistive technology, such as a new wheelchair for the care recipient, or how to safely use a transfer bench. We also assist with applications for governmental and non-profit funding programs for assistive technology or environmental modifications. We also support them in making important life decisions related to the care recipients, sometimes using decision trees or just by helping weigh the pros and cons regarding a difficult decision. Finally, we can provide emotional and moral support, as some caregivers face very serious and life-changing caregiving episodes.

There is also research work conducted in OT to support caregivers. One such example is from a team of researchers and clinicians in Quebec, led by Claudine Auger (www.movitplus.com/). The team is currently working with stakeholders to produce caregiver training and resources in relation to mobility devices. These resources come in a variety of formats, including written material, videos, and websites. They are delivered through an internet-based monitoring and support system intended to increase their access and availability.

All in all, to achieve the greatest level of care recipient quality of life, OTs work with caregivers to ensure that they can complete caregiving tasks in a manner that is safe and less burdensome for themselves and the person they care for. 

To summarize this section, we must acknowledge that caregivers contribute immensely to our society. In OT we depend on caregivers to deliver many aspects of an action plan for the care recipient. Yet, as much as we depend on caregivers to assist with such activities, caregivers also depend on OTs to help make their caregiving tasks safe, effective, and manageable. As such, one could say that, to a certain extent, it is a two-way street. We need each other. Thus, this reality is fundamental to understanding why OTs ought to approach the caregiver as a care team partner and as a client. I will now turn to further situate caregivers as a deserving and unique client population.

## Situating Caregivers as a Unique OT Client Population

As mentioned previously, many of us likely consider the caregiver when devising different courses of action for care recipient interventions or research activities. I also believe that many OTs are becoming more acutely aware of just how important caring for the caregiver is. Yet, at the present time, despite how closely we collaborate with caregivers, our work remains focused on the care recipient. That is, caregivers’ occupational needs, desires, and life goals beyond those related to a care recipient-focused intervention fall outside the scope of our work. This is often because of narrowly defined client populations outlined by jurisdictional authorities.

The point that I want to make today, however, is that approaching the caregiver as both a care collaborator and as a client will not only facilitate our efforts to improve occupational outcomes for the care recipient, but it may also produce positive outcomes for the caregiver as well. I believe we need to see caregivers as clients in their own rights who can benefit from caregiver-specific interventions. There are several ways to rationalize approaching the caregiver as an OT client emerging from research about OT values and occupational theory.

### From the Perspective of OT Values

At the moment, to the best of my knowledge, other than a brief mention in the Canadian Association of Occupational Therapists’ code of ethics, there is no nationally recognized set of OT values. So, when I say OT values, I am drawing from a 2015 study by Drolet and Désormeaux-Moreau that examined the values of OTs in Quebec (Drolet and Désormeaux-Moreau, 2015). In this study, the top three values identified by participants were: first, “autonomy”, which was defined as functional autonomy and the autonomy to freely make existential and life project decisions; second, human dignity, which the authors characterized as respecting and honoring the “clients’ subjectivity” and working with clients to help them realize “significant and signifying life projects”; and third, occupational participation, which was described as enabling individuals and communities to achieve important and meaningful occupations.

The aforementioned values strengthen and justify a case for including the caregiver within our client population. From the perspective of autonomy, it could be argued that the autonomy of the care recipient should not come at the expense of or fully infringe upon that of the caregiver. The second value, human dignity, supports the notion that OTs can work with the caregiver to assess their needs, desires, and life goals in relation to their caregiving duties and their other occupations. Finally, the third value, occupational participation, further strengthens the idea that a caregiver can, and should be, a client. Like Karen Whalley Hammell noted during her Muriel Driver lecture in 2017, OTs must “advance the right of all people to engage in occupations that contribute positively to their own well-being and the well-being of their communities” (Hammell, 2017, p. 216). For me, this includes all forms of occupations for the caregiver.

### From the Perspective of an Emerging OT Model

In addition to OT values, recent changes in OT education and conceptual framing also lend themselves to the argument that caregivers deserve to be considered as people receiving OT services in their own right. Within the next few months, *Promoting occupational participation: Collaborative, relationship-focused OT, the ‘sequel’ to Enabling Occupation* (Townsend & Polatajko, 2013) is scheduled to be published. It includes many new updates and revisions. One of the most significant changes in this edition is the new Canadian Model of Occupational Participation (Restall et al., 2021). The model shifts the clinician’s focus away from a single client-centered approach toward a focus on occupational participation.

In this approach, a single person separated into performance components is not the center of OT practice. Rather, meaningful occupational participation realized through relationships becomes the fundamental motivation for OT practice, regardless of who the person is, be they a care-recipient or caregiver. This shift in approach enhances the possibility of working with caregivers as both care collaborators and as clients by guiding OTs to explore a caregivers’ occupational participation. It allows clinicians to move beyond individualistic approaches, towards broad considerations of occupations across many different populations, scales, and contexts, including individuals, dyads, families, and communities. Based on this perspective, caregivers can be viewed as clients with important occupational needs, goals, and desires that are related to caregiving as well as other facets of their lives.

In sum, recent research and theory around OT values and practice opens the door for a new approach to caregivers as care team collaborators and as clients. By focusing on occupational participation, OTs can create holistic interventions that address the autonomy of all parties involved. We can work with caregivers and care recipients to identify signifying life projects, care-related and not, and co-strategize to achieve balance and success within them.

## Conceptualizing a Caregiver-Specific Approach

The next section will examine how we currently tend to conceptualize the caregiver experience of occupation and how this can and should be further elaborated.

### Caregiver Burden

Current education and research often root our understanding of the caregiver’s experience as burdensome and stressful. The literature describes how caregivers can experience higher rates of injury (Hinojosa & Rittman, 2009), illness (Vitaliano et al., 2003), and depression (Pinquart & Sörensen, 2003). Caregiving can lead to strained and even abusive relationships with the care recipient (Éthier et al., 2020) or add stress to relationships with family, friends (Keating & Eales, 2017), and romantic partners (Drummond et al., 2013). Additionally, caregivers report struggling to meet their employer’s expectations while also fulfilling caregiving duties, leading many to quit their jobs (Bauer & Sousa-Poza, 2015).

Much of my own research is also founded on this perspective. For example, in 2009, I led a team of researchers who developed and validated a conceptual framework regarding outcomes for caregivers of assistive technology users (Demers et al., 2009). The model was based on a stress process model of caregiving (Pearlin et al., 1990) which considers interrelated primary and secondary stressors, alongside several moderating and mediating factors. The advantage of a framework rooted in the stress process model was that it allowed us to identify and consider a variety of concerns that caregivers face and that could be alleviated with assistive technology (Demers et al., 2009). Yet, while the negative effects of caregiving are crucial to our understanding of the caregiving experience, I have come to believe our theoretical conceptualization needs to acknowledge the less considered positive effects of caregiving too.

### Positive Effects of Caregiving

Two years ago, our AGEWELL team conducted a scoping review of recent literature to identify and understand the nature of the positive effects of caregiving. We believed that an understanding of the positive aspects of caregiving could help assistive technology developers create technologies that could amplify these positive aspects (Pysklywec et al., 2020).

After reviewing 22 studies published between 2000 and 2018, we learned that caregivers report a wide range of positive effects of caregiving, including enriched relationships between the caregivers and care recipients, feelings of personal accomplishment, a sense of purpose (Li & Loke, 2013; Yu, Cheng & Wang, 2018), a sense of mastery of caregiving tasks (Lloyd et al., 2016), and improved relationships with family or others (Lloyd et al., 2016; Yu et al., 2018).

Building on this work, we envisioned a framework that situates the positive effects of caregiving as relational in nature. We identified three different types of relationalities. First, the positive effects of caregiving can be found in the relationship with oneself. Several studies reported that caregivers perceive positive effects as becoming adaptive to their “new normal,” as experiencing personal or spiritual growth, and becoming knowledgeable about care-related subject matters and skills. Several studies also noted that caregivers felt a new sense of purpose in their lives.

For example, Ribeiro and Paul (2008, p. 172) documented one caregiver’s sentiment when he spoke about caring for his spouse. He stated “I feel honoured … washing her clothes, putting them to dry, I feel honoured.… I feel an enormous pleasure in changing her, turning her, providing her with everything she needs, giving her a bath whenever needed.… I feel pleased, with [a sense of] satisfaction.” In my own experience, I too can attest to having had a renewed sense of purpose in life when I was caring for my father-in-law with my partner. I felt great satisfaction when I cooked for Hubert and saw that his appetite returned. Through caring for Hubert, many mundane everyday actions took on new meaning, which provided me with a sense of fulfillment.

The second relational type of positive effects stems from relationships with the care recipient. Caregivers noted in many studies that they gained a deepened relationship with the care recipient. For example, one person caring for their spouse with dementia described to Netto et al. (2009, p. 254) that they had “drawn closer to him. “There’s that closeness ‘cause I pay so much attention to him […] and understand him more, you know, his needs, all his needs.” Caregivers also noted that receiving praise from the care recipient for their effort was also a positive effect. As I mentioned earlier, Hubert expressed his gratitude for our caregiving, and he complimented us on our efforts, which I found to be encouraging and affirming.

Finally, caregivers noted that positive effects were also derived from relationships with others. These relationships included support and recognition from friends, family, and neighbors, or forging new relationships with other caregivers and care professionals. For example, in a study conducted by Mehrotra and Sukumar (2007), caregivers noted that new relationships with medical staff provided the caregiver with a sense of being cared for and this was interpreted as a positive effect that emerged from their caregiving experiences. From my personal experience, by overcoming the challenges and relishing in the positive effects of caregiving together, my partner and I grew and learned, and this impacted our relationship positively.

Thus, a first step to working with caregivers as clients, is to build an evidence-based understanding of the general caregiving experience. One that recognizes that caregiving episodes are stressful and burdensome but that they can also produce positive effects. Based on a review of the literature, my personal experience, and interactions with other caregivers, I believe there are positive effects, as well. These positive effects are rooted in relation to oneself, the care recipient, and with others as summarized in Table 1. This holistic understanding of the caregiver experience can lay the groundwork for a caregiver-specific approach aimed at mitigating the negative effects of caregiving, while accentuating the positive ones.

**Table 1.** Framework Situating the Positive Effects of Caregiving as Relational in Nature.

Types of relationality		Expressions
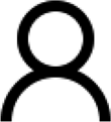	In relation to one’s self Discovering inner strengths, new areas of fulfillment, personal growth, life purpose, and/or a new or deeper sense of spirituality	○ Growing in character ○ Developing new skills and knowledge ○ Gaining confidence and competence ○ Finding a new purpose ○ Adapting to a “new normal” ○ Becoming/enhancing spirituality
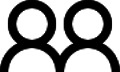	In relation to the care recipient Becoming closer to the care recipient and deriving pleasure from feeling needed and seeing the care recipient happy and comfortable	○ Receiving recognition ○ Strengthening bonds ○ Paying back/reciprocating ○ Feeling satisfied
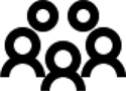	In relation to others Developing new or deeper relationships with other people. Two types of relationships dominate: family and friends; other care-related relationships (health professionals, caregivers)	○ Strengthening kinship ties ○ Finding or strengthening friendships ○ Creating relationships with health professionals ○ Connecting with other caregivers

## What a Caregiver-Specific Approach Might Look Like: A Care Scenario

To illustrate how a practitioner may go about working with a caregiver as a client, I am going to set up a care scenario loosely based on the story of a caregiver I met during a technology development workshop. Mary is a caregiver to her husband Francois, who is living at home with mobility and cognitive decline. In addition to caregiving, Mary loves gardening and teaches at a small community college. When she has the time, she volunteers for community-based transportation and nutrition programs for older adults, and before Francois’s care demanded more of her, she used to attend a book club.

As the first step in this approach, we would do what we already do, discuss with Mary her caregiving occupation. We’d work collaboratively with her to identify challenging caregiving tasks and devise ways to make them easier. The challenges that Mary mentioned included transfers, bathing, toileting, and dressing. Thus, an OT could have Mary demonstrate how she performs these activities and then offer training or other solutions to make these tasks easier.

As a second step, a practitioner could go deeper with the caregiver to learn more about him or her with respect to caregiving. This could include asking questions about motivations for caregiving (e.g., duty, accomplishment, learning, commitment). What activities or practices sustain caregiving, and what are the parts of caregiving that are enjoyable, if any? Mary revealed that she drew strength from seeing Francois comfortable and enjoyed making him nutritious meals. She also noted that she used gardening to help release the stresses associated with caregiving. Finally, she also noted that she took pride in her ability to overcome care-related challenges and enjoyed sharing her lived experience with others.

After having gained further knowledge about the challenges and positive aspects of caregiving, the last step could be to explore some of the caregivers’ needs, goals, and desires beyond caregiving. For Mary, she wanted to make time for her volunteer activities and hoped to draw on her skills as a teacher to help others learn from her caregiving experience. Taking all these aspects of Mary’s experience into consideration, an OT could potentially help Mary strategize ways to achieve these goals. With the insight that Mary feels a sense of pride for overcoming care-related challenges, and knowing that she wants to share her story, encouraging her to seek out formal or informal opportunities to discuss her caregiving journey could support her sense of occupational satisfaction. Seeking out and engaging in such activities could strengthen the positive aspects of caregiving, further sustaining her caregiving efforts, as well as provide Mary with increased social activities.

Thus, by considering both the positive and the negative aspects of caregiving, as well as the occupational needs, goals, and desires of caregivers in relation to all domains of their lives, we get a multidimensional perspective of the caregiver. With this knowledge, we can work with caregivers-as-clients to enhance their autonomy and social participation as their life circumstances change. This novel approach, with its three steps, is presented in Table 2.

**Table 2.** Steps to Caregiver-Specific Approach to Occupational Participation.

Steps	Description
Step 1	Identify challenging caregiving tasks • to make them easier • to make them safer
Step 2	Delve deeper into caregivers’ experiences • motivations for caregiving • what sustains and makes caregiving enjoyable
Step 3	Explore the caregivers’ needs, goals, and desires beyond caregiving

### Concluding Thoughts

To summarize this lecture, I began by acknowledging the massive contribution caregivers make to the Canadian healthcare system and to our work as OTs. I then shifted to discussing how both OTs and caregivers need each other to ensure that the combined efforts lead to positive outcomes for the care recipients. Next, I argued that OTs must consider caregivers as a unique client population. The logic of this reasoning is supported by recent research on OT values and shifts toward an occupational participation approach when working with clients. Yet, in order to work with caregivers-as-clients, I encourage all to incorporate a more nuanced understanding of the caregiving experience. That is one that recognizes that caregiving can be burdensome, but also one that can create positive effects for the caregiver, many of which can be identified and understood through a relational lens. In addition to the positive effects of caregiving, I also urge to consider caregiver occupation writ large across all life domains. Through a three-fold approach, we can work with caregivers to locate and mitigate negative caregiving effects, discover, and build on positive effects, and further develop positive outcomes by encouraging and balancing caregiving and non-caregiving occupations.

That said, I want to make clear that I am not arguing for a doubling of workload. Rather, a more holistic approach to caregivers that explicitly considers their needs and occupations. In time, however, OTs could, and possibly should, lobby governments for a systems-change to clearly identify who is and is not considered a client.

I strongly believe a holistic understanding of the caregiver experience embedded within an occupational participation approach used to explore the multitude of caregiver and care recipient occupations could produce a “double well-being.” That is, improved quality of life for caregivers and care recipients. As OTs, we are well-equipped for the challenge of incorporating caregivers into our practice as clients, bringing to light this potential client population who deserves our attention to guide and assist them to achieve their greatest level of occupational success.

While my preoccupation with this message pertains primarily to caregivers of older adults, it is one that resonates and can be applied to all caregivers of care recipients of all ages. I encourage us all to consider caregivers in a new light moving forward both in practice and research.

## References

[bibr1-00084174221103952] Bauer J. M. Sousa-Poza A. (2015). Impacts of informal caregiving on caregiver employment, health, and family. Journal of Population Ageing, 8(3), 113–145. 10.1007/s12062-015-9116-0

[bibr2-00084174221103952] Baum C. M. Christiansen C. H. Bass J. D. (2015). The person-environment-occupation-performance (PEOP) model. In Christiansen C. H. Baum C. M. Bass J. D. (Eds.), Occupational therapy: Performance, participation, and well-being (pp. 49–56). Slack Incorporated.

[bibr3-00084174221103952] Demers L. Fuhrer M. J. Jutai J. Lenker J. Depa M. De Ruyter F. (2009). A conceptual framework of outcomes for caregivers of assistive technology users. American Journal of Physical Medicine and Rehabilitation, 88(8), 645–655. 10.1097/PHM.0b013e3181ae0e70 19620830

[bibr4-00084174221103952] Drolet M.-J. Désormeaux-Moreau D. (2015). The values of occupational therapy: Perceptions of occupational therapists in Quebec. Scandinavian Journal of Occupational Therapy, 23(4), 272–285. 10.3109/11038128.2015.1082623 27215136

[bibr5-00084174221103952] Drummond J. D. Brotman S. Silverman M. Sussman T. Orzeck P. Barylak L. Wallach I. (2013). The impact of caregiving: Older women’s experiences of sexuality and intimacy. Affilia, 28(4), 415–428. https://doi.org/10.1177%2F0886109913504154

[bibr6-00084174221103952] Eales J. Kim C. Campbell A. Fast J. (2020). Caregivers in Nova Scotia: Economic costs and contributions. Research on Aging Policies and Practice, https://rapp.ualberta.ca/wp-content/uploads/sites/49/2020/05/2020Jan-NS-Caregivers-Economic-Costs-and-Contributions-FINAL.pdf .

[bibr7-00084174221103952] Eales J. Kim C. Riopel S. Plante M. Fast J. (2020). Caregivers in Quebec: Economic costs and contributions. Research on Aging Policies and Practice, https://rapp.ualberta.ca/wp-content/uploads/sites/49/2020/05/2020-05-Quebec-Caregivers-Economic-Costs-and-Contributions-FINAL-infographic.pdf .

[bibr8-00084174221103952] Eales J. Kim C. Sereda S. Fast J. (2019). Caregivers in Alberta: Economic costs and contributions. Research on Aging Policies and Practice, https://rapp.ualberta.ca/wp-content/uploads/sites/49/2019/05/Alberta-Caregivers-Economic-Costs-and-Contributions_2019-05-08.pdf .

[bibr9-00084174221103952] Éthier S. Beaulieu M. Perroux M. Andrianova A. Fortier M. Boisclair F. Guay M. C. (2020). Favoriser la bientraitance pour que proche aidance ne rime plus avec maltraitance. Intervention, 151, 33–46.

[bibr10-00084174221103952] Evans R. L. Connis R. T. Bishop D. S. Hendricks R. D. Haselkorn J. K. (1994). Stroke: A family dilemma. Disability and Rehabilitation, 16(3), 110–118. 10.3109/09638289409166287 7919394

[bibr11-00084174221103952] Hammell K. W. (2017). Opportunities for well-being: The right to occupational engagement. Canadian Journal of Occupational Therapy, 84(4-5), 209–222. https://doi.org/10.1177%2F0008417417734831 10.1177/0008417417734831 29364714

[bibr12-00084174221103952] Harding M. Omercic S. (2020). Care counts: Caregivers in Canada, 2018. Statistics Canada. https://www150.statcan.gc.ca/n1/en/catalogue/11-627-M2020001.

[bibr13-00084174221103952] Hinojosa M. S. Rittman M. (2009). Association between health education needs and stroke caregiver injury. Journal of Aging and Health, 21(7), 1040–1058. https://doi.org/10.1177%2F0898264309344321 19773599 10.1177/0898264309344321

[bibr14-00084174221103952] Johnson S. Bacsu J. (2018). Understanding complex care for older adults within Canadian home care: A systematic literature review. Home Health Care Services Quarterly, 37(3), 232–246. 10.1080/01621424.2018.1456996 29578846

[bibr15-00084174221103952] Keating N. Eales J. (2017). Social consequences of family care of adults: A scoping review. International Journal of Care and Caring, 1(2), 153–173. 10.1332/239788217X14937990731749

[bibr16-00084174221103952] Keating N. Eales J. Funk L. Fast J. Min J. (2019). Life course trajectories of family care. International Journal of Care and Caring, 3(2), 1a47–1a63. 10.1332/239788219X15473079319309

[bibr17-00084174221103952] Kielhofner G. Posatery Burke J. (1980). A model of human occupation, part 1: Conceptual framework and content. American Journal of Occupational Therapy, 34(9), 572–581. 10.5014/ajot.34.9.572 7457553

[bibr18-00084174221103952] Li Q. Loke A. Y. (2013). The positive aspects of caregiving for cancer patients: A critical review of the literature and directions for future research. Psycho–Oncology, 22(11), 2399–2407. 10.1002/pon.3311 23712938

[bibr19-00084174221103952] Lloyd J. Patterson T. Muers J. (2016). The positive aspects of caregiving in dementia: A critical review of the qualitative literature. Dementia (Basel, Switzerland), 15(6), 1534–1561. https://doi.org/10.1177%2F1471301214564792 10.1177/1471301214564792 25547208

[bibr20-00084174221103952] Mehrotra S. Sukumar P. (2007). Sources of strength perceived by females caring for relatives diagnosed with cancer: An exploratory study from India. Supportive Care in Cancer, 15(12), 1357–1366. 10.1007/s00520-007-0256-5 17431687

[bibr21-00084174221103952] Moghimi C. (2007). Issues in caregiving: The role of occupational therapy in caregiver training. Topics in Geriatric Rehabilitation, 23(3), 269–279. 10.1097/01.TGR.0000284770.39958.79

[bibr22-00084174221103952] Netto N. R. Jenny G. Y. N. Philip Y. L. K. (2009). Growing and gaining through caring for a loved one with dementia. Dementia (Basel, Switzerland), 8(2), 245–261. https://doi.org/10.1177%2F1471301209103269

[bibr23-00084174221103952] Pearlin L. I. Mullan J. T. Semple S. J. Skaff M. M. (1990). Caregiving and the stress process: An overview of concepts and their measures. The Gerontologist, 30(5), 583–594. 10.1093/geront/30.5.583 2276631

[bibr24-00084174221103952] Pinquart M. Sörensen S. (2003). Differences between caregivers and noncaregivers in psychological health and physical health: A meta-analysis. Psychology and Aging, 18(2), 250–267. 10.1037/0882-7974.18.2.250 12825775

[bibr25-00084174221103952] Polatajko H. J. Townsend E. A. Craik J. (2007). Canadian model of occupational performance and engagement. In Townsend E. A. Polatajko H. J. (Eds.), Enabling occupation II: Advancing an occupational therapy vision for health, well-being and justice through occupation (pp. 23). CAOT Publications ACE.

[bibr26-00084174221103952] Pysklywec A. Plante M. Auger C. Mortenson W. B. Eales J. Routhier F. Demers L. (2020). The positive effects of caring for family carers of older adults: A scoping review. International Journal of Care and Caring, 4(3), 349–375. 10.1332/239788220X15925902138734

[bibr27-00084174221103952] Reinhard S. C. Friss Feinberg L. Houser A. Choula R. Evans M. (2019). Valuing the invaluable: 2019 Update, charting a path forward. AARP Public Policy Institute. https://www.aarp.org/content/dam/aarp/ppi/2019/11/valuing-the-invaluable-2019-update-charting-a-path-forward.doi.10.26419-2Fppi.00082.001.pdf. https://doi.org/10.26419-2Fppi.00082.001.

[bibr28-00084174221103952] Restall G. Egan M. Séguin J. (2021, May 16-19). The Sequel to Enabling Occupation [Session]. Canadian Association of Occupational Therapists (CAOT) Conference, Online.

[bibr29-00084174221103952] Ribeiro O. Paul C. (2008). Older male carers and the positive aspects of care. Ageing & Society, 28(2), 165–183. 10.1017/S0144686X07006460

[bibr30-00084174221103952] Statistics Canada (2020). Caregivers in Canada, 2018: Component of statistics Canada catalogue no. 11-001-X. Statistics Canada. https://www150.statcan.gc.ca/n1/en/daily-quotidien/200108/dq200108a-eng.pdf?st = w6sMA-yV.

[bibr31-00084174221103952] Townsend E. Polatajko H. (2013). Enabling occupation II: Advancing an occupational therapy vision for health, well-being, & justice through occupation (2nd ed). CAOT Publications ACE.

[bibr32-00084174221103952] Vitaliano P. P. Zhang J. Scanlan J. M. (2003). Is caregiving hazardous to one's physical health? A meta-analysis. Psychological Bulletin, 129(6), 946–972. 10.1037/0033-2909.129.6.946 14599289

[bibr33-00084174221103952] Yu D. S. F. Cheng S. T. Wang J. (2018). Unravelling positive aspects of caregiving in dementia: An integrative review of research literature. International Journal of Nursing Studies, 79, 1–26. 10.1016/j.ijnurstu.2017.10.008 29128685

