# Supplementary material for: Expanding Occupational Therapy Perspectives with Family Caregivers
Source: Can J Occup Ther. 2022 Sep 22;89(3):223–37. doi: 10.1177/00084174221103952 (PMC9511233; doi:10.1177/00084174221103952)
Supplement: Related texts

# Other: Élargir les perspectives de l’ergothérapie auprès des proches aidants

**Authors:** Louise Demers

**Keywords:** Approche basée sur les forces, cadres de référence, champ d’activité, ergothérapie, proches aidants, relations clients-professionnels

## Abstract

**Description**. Toujours présents, les proches aidants sont des collaborateurs essentiels au travail des ergothérapeutes, mais ils sont rarement l’objet même de leurs efforts. **But**. Cette conférence aborde les possibilités intéressantes découlant d’une meilleure inclusion des proches aidants dans la pratique de l’ergothérapie. **Questions clés**. Les proches aidants constituent une clientèle unique. Cet énoncé de position est soutenu par les dernières recherches sur les valeurs des ergothérapeutes et l’évolution de la profession vers une approche de participation occupationnelle. Le travail avec cette clientèle nécessite une compréhension nuancée de son expérience. L’aidance peut être un fardeau, mais elle peut aussi avoir des effets positifs, notamment dans une perspective relationnelle. **Conséquences**. Par une approche triple, les ergothérapeutes peuvent travailler avec les proches aidants pour déterminer et atténuer les effets négatifs de l’aidance, découvrir les effets positifs et construire sur leur base, puis encourager et équilibrer les activités liées à l’aidance et les autres activités afin d’en renforcer les effets positifs.

## Introduction

Au cours de ma carrière en recherche, je me suis passionnée pour deux choses, dont, premièrement, l’analyse et la mesure de différents phénomènes occupationnels pour suivre et évaluer les changements au fil du temps. Auprès de nombreuses personnes de talent, j’ai essayé de créer des outils pour mesurer différents types de concepts importants dans les domaines de l’ergothérapie et de la réadaptation. Le second domaine de recherche ayant dominé ma carrière est celui de la gérontechnologie, particulièrement la mesure des besoins relatifs aux différentes technologies d’assistance et des répercussions de celles-ci sur les utilisateurs et les personnes aidantes. Ces dernières années, j’ai eu l’honneur de codiriger un ensemble de projets avec AGEWELL, le réseau canadien axé sur les technologies et le vieillissement (https://agewell-nce.ca/). Des collaboratrices et collaborateurs toujours présents et qui jouent un rôle crucial dans le travail des ergothérapeutes ont alors retenu mon attention. Ces personnes sont souvent indispensables à notre travail, mais elles sont rarement l’objet même de nos efforts. Il s’agit des proches aidants.

Les proches aidants sont définis comme des parents, des amis ou d’autres proches qui, volontairement ou non, prodiguent des soins physiques ou du soutien affectif à une personne vivant avec une incapacité ou des problèmes de santé mentale ou physique (Keating, Eales, Funk, Fast et Min, 2019). Ces aidants exercent souvent un travail non rémunéré pour des personnes avec lesquelles ils avaient déjà une relation. La présente conférence traitera de ce que je considère comme le rôle en pleine transformation des proches aidants dans la pratique de l’ergothérapie et des possibilités intéressantes qui en découlent.

Je commencerai par aborder la question du rôle central que jouent les proches aidants dans notre travail, dans notre société et, vraisemblablement, dans nos vies personnelles. Ensuite, je proposerai de considérer les aidants comme une clientèle unique, et je montrerai comment le fait de les approcher ainsi coïncide avec les valeurs et la théorie de l’ergothérapie. J’expliquerai comment une approche considérant les aidants comme une clientèle peut aller au-delà d’une perspective axée sur le fardeau de leur tâche en considérant un aspect de leur expérience qui, à mon avis, mérite plus d’attention, soit les effets positifs de l’aidance. Enfin, je conclurai cette conférence en proposant une approche particulière pour le travail auprès des personnes aidantes, tant comme partenaires de l’équipe de soins que comme clientèle.

À la base, ma conférence est un appel aux cliniciens et aux chercheurs, que j’invite à approfondir leur compréhension des aidants et leurs interactions avec eux dans l’exercice de l’ergothérapie, dans la recherche et dans la théorie. Je nous invite tous et toutes à considérer les personnes aidantes non seulement comme des membres de l’équipe de soins jouant un rôle essentiel pour assurer l’efficacité de l’intervention, mais aussi comme faisant partie d’une clientèle de plein droit qui survit et s’épanouit dans toutes ses activités.

## L’importance des personnes aidantes

### Un exemple personnel

J’aimerais commencer par vous faire part d’un exemple tiré de mon expérience personnelle. Il s’agit de l’une des nombreuses occasions, au cours des dernières années, à avoir mis en relief pour moi la nécessité d’une nouvelle approche considérant les aidants comme une clientèle.

Il y a quelques années, la santé et la participation de mon beau-père de 88 ans, Hubert, ont commencé à décliner. Il avait déjà subi de nombreux pontages coronariens et s’adaptait à la sarcopénie, aux pertes de mémoire et d’audition ainsi qu’aux douleurs musculosquelettiques. Il avait de plus en plus de mal à cuisiner, et il s’est mis à dormir excessivement et à se désintéresser des activités sociales. Il est devenu clair qu’il nécessitait et souhaitait une aide supplémentaire pour certaines activités de la vie quotidienne. Mon conjoint et moi sommes ainsi devenus des proches aidants.

Pour moi, c’était nouveau. Je n’avais jamais tenu ce rôle pour qui que ce soit d’autre que mes propres enfants. Au fil des mois, il est devenu de plus en plus difficile de continuer à aider Hubert tout en répondant aux demandes de notre travail. Par chance, nous avons réussi à lui obtenir des services de soins à domicile pour les jours de semaine, avec entretien ménager, soins matinaux, prise de médicaments, et deux repas par jour. La fin de semaine, mon conjoint et moi prenions en charge certaines de ces tâches. Ensemble, Hubert, mon conjoint, sa famille, le personnel aidant et moi-même avons travaillé à atteindre une certaine stabilité dans nos nouvelles routines.

Pendant cet épisode d’aidance, j’ai ressenti une vaste gamme d’émotions. En semaine, mon conjoint et moi nous inquiétions au sujet d’Hubert. Tout appel imprévu nous faisait sursauter : était-ce de mauvaises nouvelles? Lorsque nous lui téléphonions et qu’il ne répondait pas, nous craignions que quelque chose se soit passé. Était-il tombé? Est-ce que ça allait? Nous étions frustrés, et parfois nous nous sentions coupables de ne pas être plus présents. Les fins de semaine, je me sentais parfois épuisée, physiquement et mentalement, par certaines des tâches, et bouleversée par le déclin continu d’Hubert. Néanmoins, j’avais approfondi mes relations avec lui ainsi qu’avec mon conjoint, et c’est là un aspect gratifiant dont j’étais reconnaissante. À la fin de sa vie, Hubert était encore capable de dire à quel point nous étions importants pour lui. Son décès à l’été 2020, vers le début de la pandémie de COVID-19, m’a profondément attristée, malgré un certain soulagement. Le fait d’avoir eu la chance d’en prendre soin et de l’entendre nous dire à quel point nos années d’aidance avaient été importantes pour lui demeure très important pour moi.

Je suis bien consciente du caractère « ordinaire » de l’histoire personnelle que je raconte ici. Mon expérience à titre de proche aidante n’a rien d’unique et elle n’a pas été particulièrement longue ou difficile, mais c’est précisément là que je veux en venir. Je vous fais part de mon expérience de proche aidante pour souligner que le fait de prendre soin d’un parent ou d’un ami est un rôle quasi universel que la plupart d’entre nous aurons à jouer dans notre vie, et fort probablement plus d’une fois, si ce n’est déjà le cas. La nature universelle de l’aidance est la principale raison pour laquelle les ergothérapeutes doivent continuer d’approfondir leur compréhension des proches aidants et leurs interactions avec ceux-ci.

### Nous sommes (ou serons) tous des aidants

Le profil et la contribution des proches aidants dans la société canadienne ont un caractère dynamique et profond. Lors du recensement de 2018, un Canadien sur quatre âgé de 15 ans ou plus rapportait s’occuper d’un parent ou d’un ami vivant avec une maladie chronique, une incapacité ou des besoins liés à l’âge (Harding et Omercic, 2020; Statistique Canada, 2020). Sur les quelque 7,8 millions de proches aidants au Canada, environ 54% s’identifiaient comme des femmes, 47% s’occupaient comme moi d’un parent ou d’un beau-parent, 13% s’occupaient d’un conjoint et 13% s’occupaient d’un ami, d’un voisin ou d’une connaissance (Harding et Omercic, 2020; Statistique Canada, 2020).

Bien évidemment, la contribution sociale et économique des proches aidants et de leur travail est importante pour la société canadienne, et particulièrement pour nos systèmes de santé. Historiquement, ceux-ci ont toujours compté sur les proches aidants partout au pays (Reinhard, Friss Feinberg, Houser, Choula et Evans, 2019), et cette dépendance ne fait que croître alors que les patients obtiennent leur congé d’hôpital de plus en plus tôt et que les aidants doivent effectuer à la maison des tâches de plus en plus complexes (Johnson et Bacsu, 2018).

Les économies que les aidants permettent aux systèmes de santé de réaliser sont inestimables. Malgré tout, certains chercheurs ont tenté de les quantifier. Au Québec, par exemple, on estime qu’il y a 1,9 million de proches aidants, qui fournissent annuellement 743 millions d’heures de travail non rémunéré. Cela permet au système de santé d’économiser environ 9,3 milliards de dollars par année ( Eales, Kim, Riopel, Plante et Fast, 2020), et on ne parle que du Québec. En Alberta, on estime que les 388 millions d’heures de travail non rémunéré effectuées annuellement par près d’un million d’aidants permettent aux Alberta Health Services d’économiser 5,8 milliards de dollars par année (Eales, Kim, Sereda et Fast, 2019). L’histoire est sensiblement la même dans toutes les provinces et tous les territoires du Canada (voir Eales, Kim, Campbell et Fast, 2020; Eales, Kim, Riopel, Plante et Fast, 2020 pour d’autres exemples). Malgré ces chiffres, très peu de personnes aidantes ont été formées pour occuper ce rôle.

Les aidants et aidantes peuvent avoir du mal à trouver un équilibre entre leurs différentes sphères d’activité – comme le travail rémunéré, les autres engagements auprès des parents et amis et de la collectivité, ainsi que les activités de loisir et de détente. Sans eux, pourtant, plusieurs des bienfaits pour les personnes bénéficiaires de soins seraient compromis, voire impossibles.

### Les ergothérapeutes ont besoin des aidants

À titre d’ergothérapeutes, nous sommes bien au fait du rôle crucial et difficile que jouent les proches aidants dans la vie des personnes ayant une maladie chronique, une incapacité ou des besoins liés à l’âge. Dès le début de notre formation, on nous apprend à comprendre la complexité des dynamiques familiales autour du patient et de ses soins (Moghimi, 2007). La personne aidante est ainsi incluse dans les modèles qui façonnent la pratique et la recherche en ergothérapie. Par exemple, le Modèle canadien du rendement et de l’engagement occupationnel considère les aidants comme l’un des quatre facteurs environnementaux qui influencent une personne et ses activités (Polatajko, Townsend et Craik, 2007). Le Modèle de l’occupation humaine situe les personnes (y compris les proches aidants) parmi les intrants et les extrants de l’environnement de la personne bénéficiaire des soins (Kielhofner et Posatery Burke, 1980). Enfin, le Modèle personne-environnement-occupation-rendement positionne également les proches aidants au sein de l’« environnement » de la personne bénéficiaire des soins à titre de soutien social, avec d’autres facteurs tels que les systèmes sociaux et économiques, la culture et les valeurs, les technologies ainsi que l’environnement bâti et naturel (Baum, Christiansen et Bass, 2015).

Je suis certaine que ces modèles trouvent un écho dans l’expérience de nombreux cliniciens et chercheurs en ergothérapie. Nous pouvons vraisemblablement tous attester du fait que les aidants constituent un facteur environnemental important ou influencent notre travail. Les aidants jouent un rôle crucial dans la mise en œuvre des recommandations qui améliorent la vie des bénéficiaires. En effet, les chercheurs ont noté que les gains réalisés lors de la réadaptation étaient maintenus de manière plus efficace lorsque les membres de la famille étaient engagés et offraient leur soutien (Evans, Connis, Bishop, Hendricks et Haselkorn, 1994).

On peut donc dire que les ergothérapeutes ont besoin des aidants pour les soutenir dans une grande variété de tâches centrales dans leur plan d’action auprès des personnes bénéficiaires de soins. Ces tâches peuvent comprendre la gestion de la médication, l’aide à la mobilité et aux transferts, la communication des besoins en matière de soins de santé, et ainsi de suite. Sans les personnes aidantes, le travail des ergothérapeutes serait vraisemblablement moins efficace.

### Les aidants ont besoin des ergothérapeutes

Réciproquement, si les ergothérapeutes ont besoin des aidants, de nombreux aidants conviendront aussi avoir besoin des ergothérapeutes. Alors que les aidants aident à atteindre les objectifs de nos interventions axées sur les bénéficiaires de soins, plusieurs de celles-ci améliorent et facilitent les soins qu’ils offrent. Par exemple, nous formons les aidants sur la manière d’effectuer leurs tâches, nous leur donnons la possibilité de mettre en pratique de nouvelles compétences liées à la prestation de soins, et nous leur offrons de la rétroaction et de l’accompagnement (Moghimi, 2007). Nous pouvons également améliorer les compétences et la littératie en matière de santé des personnes aidantes en les formant sur les technologies d’assistance telles qu’un nouveau fauteuil roulant ou sur l’utilisation sécuritaire d’un banc de transfert. Nous les aidons aussi à présenter des demandes aux programmes de financement gouvernementaux et d’organismes sans but lucratif pour les technologies d’assistance et les modifications à l’environnement. Nous les aidons à prendre des décisions de vie importantes en ce qui concerne la personne bénéficiaire des soins, parfois à l’aide d’arbres décisionnels, ou simplement en les amenant à peser les pour et les contre lors d’un choix difficile. Finalement, nous pouvons leur offrir un soutien affectif et moral, alors que certains aidants traversent des épreuves qui changent leur vie.

Des recherches sont également effectuées dans le domaine de l’ergothérapie pour soutenir les personnes aidantes. Un exemple est celui d’une équipe de chercheurs et de cliniciens québécois dirigée par Claudine Auger (www.movitplus.com/). L’équipe travaille actuellement avec les parties prenantes pour produire une formation et des ressources en lien avec les dispositifs d’aide à la mobilité. Ces ressources se présentent sous diverses formes, y compris des documents écrits, des vidéos et des sites Web. Elles sont offertes par l’intermédiaire d’un système de suivi et de soutien sur Internet, qui vise à en accroître l’accessibilité.

Dans l’ensemble, afin de maximiser la qualité de vie des bénéficiaires de soins, les ergothérapeutes travaillent avec les aidants afin de leur permettre d’exercer leurs tâches en toute sécurité et de la manière la moins lourde possible pour eux et les personnes dont ils prennent soin. Pour résumer cette section, nous devons reconnaître que les aidants contribuent énormément à notre société. En ergothérapie, nous comptons sur les aidants pour livrer plusieurs aspects du plan d’action auprès de la personne bénéficiaire des soins. Si nous dépendons d’eux pour nous aider dans ces activités, les aidants dépendent aussi des ergothérapeutes pour les aider à rendre leurs tâches sécuritaires, efficaces et gérables. Dans une certaine mesure, on peut dire que la relation est bidirectionnelle. Nous avons besoin les uns des autres. Cette réalité est fondamentale pour comprendre pourquoi les ergothérapeutes doivent considérer les aidants à la fois comme des partenaires de l’équipe de soins et comme des clients. Je vais maintenant expliquer en quoi les aidants constituent une clientèle unique qui mérite notre attention.

## Les aidants comme clientèle unique en ergothérapie

Comme je l’ai mentionné précédemment, plusieurs d’entre nous prennent vraisemblablement en compte la personne aidante au moment de déterminer différents plans d’action pour les interventions auprès des bénéficiaires de soins ou les activités de recherche. Je crois également que de nombreux ergothérapeutes sont de plus en plus conscients de l’importance de prendre soin des personnes aidantes. Pour le moment, néanmoins, même lorsque nous collaborons de près avec les personnes aidantes, notre travail demeure axé sur les bénéficiaires des soins. C’est-à-dire que les objectifs de vie, les désirs et les besoins occupationnels des personnes aidantes allant au-delà de ceux qui sont liés à une intervention axée sur les personnes bénéficiaires des soins sortent de notre cadre de travail. Cela s’explique souvent par le fait que nos clientèles sont définies étroitement par les autorités compétentes.

L’idée que je veux défendre aujourd’hui, toutefois, est que le fait de considérer les aidants à la fois comme des collaborateurs et comme des clients faciliterait nos efforts pour améliorer les résultats occupationnels, tant pour les bénéficiaires des soins que pour les aidants. Je crois que nous devons considérer les personnes aidantes comme des clients de plein droit pouvant bénéficier d’interventions leur étant spécifiquement destinées. Il y a diverses manières de rationaliser l’approche qui émerge des recherches sur les valeurs de l’ergothérapie et la théorie occupationnelle à l’égard de la clientèle des aidants.

### Dans la perspective des valeurs de l’ergothérapie

Pour le moment, il n’existe à ma connaissance, outre une brève mention dans le code d’éthique de l’Association canadienne des ergothérapeutes (ACE), aucun ensemble de valeurs de l’ergothérapie reconnu à l’échelle nationale. Lorsque je parle des valeurs de l’ergothérapie, je fais donc référence à une étude de 2015 de Drolet et Désormeaux-Moreau se penchant sur les valeurs de l’ergothérapie au Québec. Dans cette étude, les trois principales valeurs désignées par les participants étaient, dans l’ordre, l’autonomie, comprise comme l’autonomie fonctionnelle et le fait de pouvoir prendre librement des décisions existentielles et liées aux projets de vie; la dignité humaine, caractérisée comme le respect de la subjectivité et le fait de travailler avec les clients pour les aider à réaliser des projets de vie significatifs; et enfin la participation occupationnelle, décrite comme le fait de permettre aux individus et aux collectivités de réaliser des occupations importantes et significatives.

Les valeurs susmentionnées renforcent et justifient l’inclusion de la personne aidante parmi notre clientèle. Pour ce qui est de l’autonomie, on pourrait faire valoir que l’atteinte de l’autonomie de la personne bénéficiaire des soins ne devrait pas nuire à celle de l’aidant ou empiéter entièrement sur celle-ci. La deuxième valeur, celle de la dignité humaine, soutient l’idée selon laquelle les ergothérapeutes peuvent travailler avec la personne aidante pour évaluer ses besoins, ses désirs et ses objectifs de vie en lien avec ses tâches d’aidance et ses autres activités. Enfin, la troisième valeur, celle de la participation occupationnelle, renforce encore davantage l’idée selon laquelle les personnes aidantes peuvent, et doivent, être incluses dans notre clientèle. Comme l’a noté Karen Whalley Hammell dans son Discours commémoratif Muriel Driver en 2017, les ergothérapeutes doivent faire valoir « le droit de toute personne à s’engager dans des occupations signifiantes qui contribuent positivement à leur bien-être personnel et au bien-être de leurs communautés » (Hammell, 2017, p. 216). Pour moi, cela inclut toutes les activités de la personne aidante.

### Dans la perspective d’un modèle émergent pour l’ergothérapie

En plus des valeurs de l’ergothérapie, les changements récents dans la formation et le cadre conceptuel de la profession tendent vers l’idée selon laquelle les aidants méritent d’être considérés comme des bénéficiaires de plein droit des services d’ergothérapie. Dans quelques mois paraîtra *Promoting occupational participation : Collaborative, relationship-focused OT*, la « suite » d’*Habiliter à l’occupation* (Townsend et Polatajko, 2013), avec de nombreuses révisions et mises à jour. L’un des principaux changements dans cette édition sera le nouveau Modèle canadien de la participation occupationnelle (Restall, Egan et Séguin, 2021). Ce modèle remplace l’approche clinique centrée sur un seul client par une approche axée sur la participation occupationnelle.

Selon celle-ci, l’exercice de l’ergothérapie n’est pas axé sur un individu séparé en composantes du rendement. C’est plutôt une participation occupationnelle significative réalisée à travers des relations qui devient la motivation fondamentale de l’exercice de l’ergothérapie, et ce, que la personne soit bénéficiaire de soins ou aidante. Ce changement d’approche met en valeur la possibilité de travailler avec les aidants tant à titre de collaborateurs que de clients en guidant les ergothérapeutes dans l’exploration de la participation occupationnelle de l’aidant. Elle permet aux cliniciens d’aller au-delà des approches individualistes et vers une considération plus vaste des occupations à travers différentes populations, différentes échelles et différents contextes incluant les individus, les dyades, les familles et les communautés. Dans cette perspective, les aidants peuvent être vus comme des clients avec d’importants besoins occupationnels, des objectifs et des désirs liés à l’aidance comme aux autres facettes de leur vie.

En somme, les récentes recherches et théories autour des valeurs et de l’exercice de l’ergothérapie ouvrent la porte à une nouvelle approche considérant les aidants à la fois comme des collaborateurs et comme des clients. En se concentrant sur la participation occupationnelle, les ergothérapeutes peuvent créer des interventions holistiques favorisant l’autonomie de toutes les parties. Nous pouvons travailler avec les aidants et les bénéficiaires de soins pour trouver des projets de vie significatifs en lien avec les soins et non liés à ceux-ci, et trouver ensemble des stratégies pour y réussir et trouver un équilibre entre eux.

## Conceptualiser une approche particulière aux aidants

Dans la prochaine section, je parlerai de la manière dont nous tendons à conceptualiser l’expérience occupationnelle de la personne aidante et de la manière dont celle-ci peut et doit être développée davantage.

### Le fardeau des personnes aidantes

La formation et la recherche actuelles tendent à nous faire concevoir l’expérience de la personne aidante comme un fardeau et une source de stress. La littérature démontre comment les aidants sont plus vulnérables aux blessures (Hinojosa et Rittman, 2009), à la maladie (Vitaliano, Zhang et Scanlan, 2003) et à la dépression (Pinquart et Sörensen, 2003). L’aidance peut donner lieu à des relations difficiles et parfois même empreintes de violence avec la personne bénéficiaire des soins (Éthier, Beaulieu, Perroux, Andrianova, Fortier, Boisclair et Guay, 2020) ou ajouter de la tension dans les relations avec la famille et les amis (Keating et Eales, 2017) ainsi qu’avec les conjoints (Drummond, Brotman, Silverman, Sussman, Orzeck, Barylak et Wallach, 2013). En outre, les aidants signalent avoir du mal à répondre aux attentes de leur employeur tout en exerçant leurs tâches d’aidance, ce qui en amène plusieurs à quitter leur emploi (Bauer et Sousa-Poza, 2015).

Beaucoup de mes propres recherches sont également fondées sur cette perspective. En 2009, par exemple, j’ai dirigé une équipe de chercheurs qui avait élaboré et validé un cadre conceptuel au sujet des répercussions, pour les aidants, du recours aux technologies d’assistance (Demers, Fuhrer, Jutai, Lenker, Depa et De Ruyter, 2009). Celui-ci était basé sur un modèle des processus de stress associés à l’aidance (Pearlin, Mullan, Shierly, Semple et Skaff, 1990), considérant les stresseurs primaires et secondaires interreliés parallèlement à plusieurs facteurs de modération et de médiation. L’avantage d’un cadre ancré dans le modèle des processus de stress était de permettre d’identifier et de considérer une variété d’inquiétudes auxquelles les aidants font face, et que les technologies d’assistance peuvent aider à réduire (Demers et al., 2009). Néanmoins, si la prise en compte des effets négatifs est cruciale pour comprendre l’expérience de l’aidance, j’en suis venue à croire que la conceptualisation théorique devait également reconnaître les effets positifs de l’aidance, qui sont plus souvent laissés de côté.

### Les effets positifs de l’aidance

Il y a deux ans, notre équipe à AGEWELL a réalisé une étude de portée de la littérature récente pour déterminer les effets positifs de l’aidance et en comprendre la nature. Nous étions d’avis qu’une compréhension des aspects positifs de l’aidance pourrait aider les concepteurs à créer des technologies d’assistance aptes à les amplifier (Pysklywec, Plante, Auger, Mortenson, Eales, Routhier et Demers, 2020).

Après avoir passé en revue 22 études publiées entre 2000 et 2018, nous avons appris que les aidants faisaient état d’une vaste gamme d’effets positifs de l’aidance, dont l’approfondissement de la relation entre l’aidant et la personne bénéficiaire des soins, un sentiment de réalisation personnelle et de raison d’être (Li et Loke, 2013; Yu, Cheng et Wang, 2018), un sentiment de maîtrise des tâches d’aidance (Lloyd et al., 2016), et une amélioration des relations avec la famille ou les autres (Lloyd, Patterson et Muers, 2016; Yu et al., 2018).

Sur la base de ce travail, nous avons envisagé un cadre situant les effets positifs de l’aidance comme étant relationnels par nature. Nous avons défini trois types de relationnalité. Tout d’abord, les effets positifs de l’aidance peuvent se faire ressentir dans la relation avec soi. De nombreuses études rapportent que les aidants perçoivent des effets positifs tels que le fait d’avoir su s’adapter à leur « nouvelle normalité », de se sentir grandir sur le plan personnel ou spirituel et de développer des connaissances et des compétences sur les questions relatives aux soins. Plusieurs études ont également montré que des personnes aidantes se découvraient une nouvelle raison d’être.

Par exemple, Ribeiro et Paul (2008, p. 172) ont documenté les sentiments d’un aidant à l’égard des soins qu’il offrait à son épouse. Il disait : « Je me sens honoré […] de laver et de faire sécher ses vêtements […]. J’ai beaucoup de plaisir à la changer, à la tourner, à lui donner tout ce dont elle a besoin, à lui donner son bain chaque fois qu’elle en a besoin […] Je suis ravi, [j’en éprouve de la] satisfaction ». Je peux témoigner moi aussi avoir ressenti une nouvelle raison d’être lorsque je me suis occupée de mon beau-père avec mon conjoint. J’ai trouvé très satisfaisant de cuisiner pour Hubert et de voir son appétit revenir. En m’occupant d’Hubert, de nombreuses actions de tous les jours ont pris pour moi une nouvelle signification, ce qui m’a donné un sentiment de plénitude.

Le deuxième type d’effets relationnels positifs découle des relations avec la personne bénéficiaire des soins. Dans de nombreuses études, les aidants indiquent avoir approfondi leur relation avec la personne bénéficiaire. Par exemple, une personne qui s’occupe de son conjoint atteint de démence a expliqué à Netto, Jenny et Philip (2009, p. 254) s’en être « rapprochée. Il y a cette proximité, parce que je lui accorde tellement d’attention […] et je le comprends mieux, avec ses besoins, tous ses besoins. » Des aidants disent également que les remerciements de la personne bénéficiaire constituaient un effet positif. Comme je l’ai mentionné précédemment, Hubert nous exprimait sa gratitude pour nos soins et nous complimentait sur nos efforts, ce que j’ai trouvé encourageant.

Enfin, des aidants indiquent éprouver des effets positifs grâce à leurs relations avec les autres. Ces relations comprennent le soutien et la reconnaissance des amis, parents et voisins, et l’établissement de nouveaux liens avec les autres aidants et les professionnels. Par exemple, dans une étude réalisée par Mehrotra et Sukumar (2007), des aidants soulignent que de nouvelles relations avec le personnel médical leur ont donné le sentiment que l’on prenait soin d’eux, ce qu’ils interprètent comme un effet positif de leur expérience d’aidance. Dans ma propre expérience, le fait d’avoir surmonté les défis et apprécié ensemble les effets positifs de l’aidance nous a permis, à mon conjoint et moi, de grandir et d’apprendre, ce qui a eu des répercussions positives sur notre relation.

Ainsi, une première étape du travail avec les aidants comme clientèle consiste à établir une compréhension de l’expérience générale de l’aidance sur la base de données probantes. Il s’agit de reconnaître le fait que les épisodes d’aidance sont stressants et lourds, mais qu’ils peuvent aussi produire des effets positifs. C’est ce que je crois également, sur la base d’une revue de la littérature, de mon expérience personnelle et de mes interactions avec d’autres personnes aidantes. Ces effets positifs sont ancrés dans la relation avec soi, avec la personne bénéficiaire des soins et avec les autres, comme le résume le tableau 1. Cette compréhension holistique de l’expérience de la personne aidante peut jeter les bases d’une approche axée sur les aidants et visant à atténuer les effets négatifs de l’aidance, tout en accentuant les effets positifs.

## Proposition de scénario d’une approche particulière aux aidants

Pour illustrer la manière dont un praticien peut travailler avec la clientèle des personnes aidantes, je vais établir un scénario de soins vaguement basé sur l’histoire d’une aidante que j’ai rencontrée lors d’un atelier de développement de technologie. Marie est la proche aidante de son conjoint François, qui vit à la maison et dont la mobilité et les facultés cognitives déclinent. En plus de ses tâches d’aidante, Marie aime jardiner, et elle enseigne dans un collège communautaire. Lorsqu’elle en a le temps, elle fait du bénévolat pour des programmes communautaires de transport et de nutrition destinés aux personnes âgées, et avant de devoir consacrer plus de temps aux soins de François, elle faisait partie d’un club de lecture.

Comme première étape de notre approche auprès des personnes aidantes, nous ferions avec Marie ce que nous faisons déjà, à savoir, discuter de ses activités d’aidance. Nous travaillerions en collaboration pour cibler les tâches qui lui posent des difficultés, et trouver des manières de les faciliter. En l’occurrence, les difficultés mentionnées par Marie comprenaient les transferts, le bain, l’aide pour aller à la toilette et l’habillement. Sachant cela, l’ergothérapeute lui demanderait de lui montrer comment elle effectue ces tâches, et lui offrirait de la formation ou d’autres solutions pour aider à les faciliter.

Dans un deuxième temps, le praticien pourrait essayer d’aller un peu plus loin pour en apprendre davantage sur la personne aidante, en lien avec l’aidance. Il pourrait par exemple la questionner sur ce qui la motive à être une proche aidante (p. ex. : sentiment de responsabilité, accomplissement, apprentissage, engagement). Quelles sont les activités ou les pratiques qui soutiennent les soins, et quels sont les aspects de l’aidance qui lui sont plaisants, le cas échéant? Marie a révélé qu’elle puisait des forces dans le fait de voir François à l’aise, et qu’elle aimait lui préparer des repas nutritifs. Elle a également mentionné se servir du jardinage comme moyen de réduire le stress associé à l’aidance. Enfin, elle a dit tirer de la fierté du fait de pouvoir relever des défis liés aux soins et aimer pouvoir témoigner de son expérience avec d’autres.

Après en avoir appris davantage sur les défis et les aspects positifs de l’aidance, la dernière étape pourrait être d’explorer certains des besoins, des objectifs et des désirs des personnes aidantes, au-delà de l’aidance. Marie, pour sa part, voulait trouver du temps pour ses activités bénévoles, et espérait pouvoir mettre à profit ses compétences en enseignement pour aider les autres à apprendre de son expérience d’aidance. En prenant en compte tous ces aspects de l’expérience de Marie, un ergothérapeute pourrait l’aider à élaborer des stratégies pour atteindre ces objectifs. En sachant que Marie tire de la fierté du fait de relever des défis liés aux soins et qu’elle veut faire part de son histoire aux autres, le fait de l’encourager à rechercher des occasions officielles ou non officielles de discuter de son parcours pourrait contribuer à soutenir son sentiment de satisfaction occupationnelle. La recherche de telles activités et l’engagement dans celles-ci pourraient renforcer les aspects positifs de l’aidance et soutenir davantage les efforts de Marie dans ce sens, en plus de lui donner accès à plus d’activités sociales.

Ainsi, en considérant les aspects tant positifs que négatifs de l’aidance, de même que les désirs, les objectifs et les besoins occupationnels des aidants en lien avec toutes les sphères de leur vie, nous obtenons une perspective multidimensionnelle de la personne aidante. Avec ce savoir, nous pouvons travailler avec la clientèle des aidants pour augmenter leur autonomie et leur participation sociale en fonction de l’évolution de leurs circonstances de vie. Cette nouvelle approche en trois étapes est présentée au tableau 2.

## Réflexions en guise de conclusion

Pour résumer, j’ai commencé cette conférence en reconnaissant l’immense contribution des proches aidants au système de santé canadien et à notre travail comme ergothérapeutes. J’ai ensuite abordé le fait que tant les ergothérapeutes que les personnes aidantes avaient besoin les uns des autres pour veiller à ce que leurs efforts combinés entraînent des effets positifs pour les bénéficiaires de soins, puis j’ai soutenu que les ergothérapeutes devaient considérer les aidants comme une clientèle unique. La logique derrière ce raisonnement est soutenue par les dernières recherches sur les valeurs de l’ergothérapie, et le tournant vers une approche axée sur la participation occupationnelle lors du travail avec la clientèle. Néanmoins, pour travailler avec la clientèle des aidants, je nous encourage tous et toutes à intégrer une compréhension plus nuancée de l’expérience de l’aidance. C’est-à-dire une approche qui reconnaît que l’aidance peut être un fardeau, mais qu’elle peut aussi avoir des effets positifs pour les aidants, notamment dans une perspective relationnelle. En plus des effets positifs de l’aidance, je nous invite fortement à considérer l’occupation d’aidant ou d’aidante dans toutes les sphères de la vie. Par une approche triple, nous pouvons travailler avec les proches aidants pour déterminer et atténuer les effets négatifs de l’aidance, découvrir les effets positifs et construire sur leur base, puis encourager et équilibrer les activités liées à l’aidance et les autres activités afin d’en renforcer les effets positifs.

Cela étant dit, je veux qu’il soit clair que je ne souhaite pas doubler ainsi notre charge de travail. Je fais plutôt la promotion d’une approche plus holistique abordant explicitement les besoins et les occupations des personnes aidantes. Avec le temps, néanmoins, les ergothérapeutes pourraient, et devraient sans doute, faire pression après des gouvernements pour changer le système de manière à identifier clairement qui est un client et qui ne l’est pas.

Je crois fortement qu’une compréhension globale de l’expérience de la personne aidante, ancrée dans une approche de participation occupationnelle et visant à explorer la multitude d’occupations des aidants et des bénéficiaires de soins, pourrait produire un « double bien-être ». Autrement dit, une meilleure qualité de vie pour les aidants et pour les bénéficiaires de soins. Comme ergothérapeutes, nous sommes bien équipés pour relever le défi de travailler auprès des personnes aidantes, mettant en lumière cette clientèle potentielle qui mérite notre attention de manière à la guider et à l’aider à s’épanouir sur le plan occupationnel.

Si, en livrant ce message, je pense d’abord aux aidants de personnes âgées, celui-ci peut trouver un écho ailleurs et s’appliquer aux aidants qui œuvrent auprès de personnes de tous âges. Je nous encourage tous et toutes à considérer les aidants sous un nouveau jour, tant dans l’exercice de notre profession que dans la recherche.
